# Regulatory Role of Retinoic Acid in Male Pregnancy of the Seahorse

**DOI:** 10.1016/j.xinn.2020.100052

**Published:** 2020-10-13

**Authors:** Chunyan Li, Yongxin Li, Geng Qin, Zelin Chen, Meng Qu, Bo Zhang, Xue Han, Xin Wang, Pei-yuan Qian, Qiang Lin

**Affiliations:** 1 CAS Key Laboratory of Tropical Marine Bio-Resources and Ecology, South China Sea Institute of Oceanology, Innovation of South China Sea Ecology and Environmental Engineering, Chinese Academy of Sciences, 510301 Guangzhou, China; 2 Southern Marine Science and Engineering Guangdong Laboratory (Guangzhou), 511458 Guangzhou, China; 3 Department of Chemistry, The University of Hong Kong, Pokfulam, Hong Kong, Hong Kong SAR, China; 4 Department of Ocean Science and Hong Kong Branch of Southern Marine Science and Engineering Guangdong Laboratory (Guangzhou), Hong Kong University of Science and Technology, Kowloon, Hong Kong, China

**Keywords:** seahorse, male pregnancy, brood pouch, integrative omics, retinoic acid, antioxidant defense

## Abstract

Seahorses epitomize the exuberance of evolution. They have the unique characteristic of male pregnancy, which includes the carrying of many embryos in a brood pouch that incubates and nourishes the embryos, similar to the mammalian placenta. However, the regulatory networks underlying brood pouch formation and pregnancy remain largely unknown. In this study, comparative transcriptomic and metabolomic profiling on the lined seahorse *Hippocampus erectus*, with unformed, newly formed, and pregnant brood pouches identified a total of 141 and 2,533 differentially expressed genes together with 73 and 121 significantly differential metabolites related to brood pouch formation and pregnancy, respectively. Specifically, integrative omics analysis revealed that retinoic acid (RA) synthesis and signaling pathway played essential roles in the formation of the brood pouch and pregnancy. RA might function upstream of testosterone and progesterone, thereby directly influencing brood pouch formation by regulating the expression of *fshr* and *cyp7a1*. Our results also revealed that RA regulates antioxidant defenses, particularly during male pregnancy. Alternatively, pregnancy caused a consistent decrease in RA, canthaxanthin, astaxanthin, and glutathione synthetase, and an increase in susceptibility to oxidative stress, which may balance brood pouch development and reproduction in seahorses and pave the way to successful gestation.

## Public Summary

• Male seahorses epitomize the unique characteristic of male pregnancy
• Retinoic acid (RA) plays key regulatory roles in brood pouch formation and pregnancy
• RA could influence the brood pouch formation by regulating steroid hormone metabolism
• RA may contribute to the successful gestation by regulating antioxidant defense

## Graphical Abstract

**Figure:**
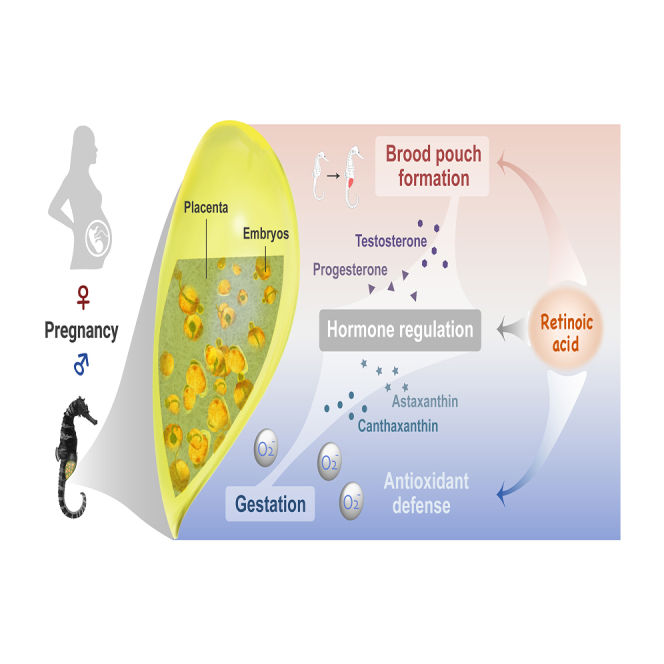

## Introduction

Syngnathid fish possess the unique reproductive strategy of male pregnancy, in which the male possesses an embryo-incubating structure called a brood pouch.^1^^,^^2^ Both the structure and position of the brood pouch vary substantially among syngnathids and pouch ultrastructure and functions differ even among closely related species.^3^ Among syngnathid fish, seahorses (*Hippocampus*) not only have the most complex pouch structure but also experience the most significant physiological changes during embryo incubation.^4^ A brood pouch is not present in juvenile seahorses but rather appears with growth in male seahorses.^5^ Male seahorses first form a baggy structure from the primordium, followed by differentiation and establishment of brood pouch-specific tissues, which ultimately form a pouch with well-developed blood vessels capable of incubating embryos.^2^ Seahorses have placenta-like tissues that facilitate pregnancy, which are similar to the placental structure in mammals.^4^ Moreover, the significant morphological and functional changes of the brood pouch that occur during gestation are equivalent to those in the uterus of the mammals.^6^ For example, it has been suggested that heavily vascularized tissues in the brood pouch are related to gas exchange between the embryos and the father,^7^ while abundant C-type lectins present during early incubation may have an important immune protective role for developing embryos.^8^ Although a few studies have sought to identify specific genes and pathways associated with pouch function,^9^^,^^10^ a comprehensive analysis of the regulatory gene and metabolite networks responsible for control of brood pouch formation and pregnancy has not yet been conducted.

Retinoic acid (RA) has multiple functions in a wide range of biological processes, particularly in early organogenesis induction and patterning,^11^, ^12^, ^13^, ^14^ as well as in innate and adaptive immunity.^15^ RA receptors are involved in the regulation of androgen biosynthesis,^16^^,^^17^ which is necessary for the production of seahorse pouches.^4^ Due to the pleiotropic roles of RA in the immune system,^15^ any deviation from the required concentration of RA can result in oxidative stress, while impacting normal growth and differentiation.^18^ However, the effects of RA signaling in syngnathids remain unclear despite the diverse brooding types and structures present in this family. In this study, we aimed to investigate the molecular mechanism underlying brood pouch development by combining both transcriptomic and metabolomic analyses of the lined seahorse (*Hippocampus erectus*) at different pouch developmental stages with specific focus on the regulatory role of RA in brood pouch formation and pregnancy.

## Results

### Changes in Genes and Metabolites during Brood Pouch Formation and Pregnancy

**Figure 1:**
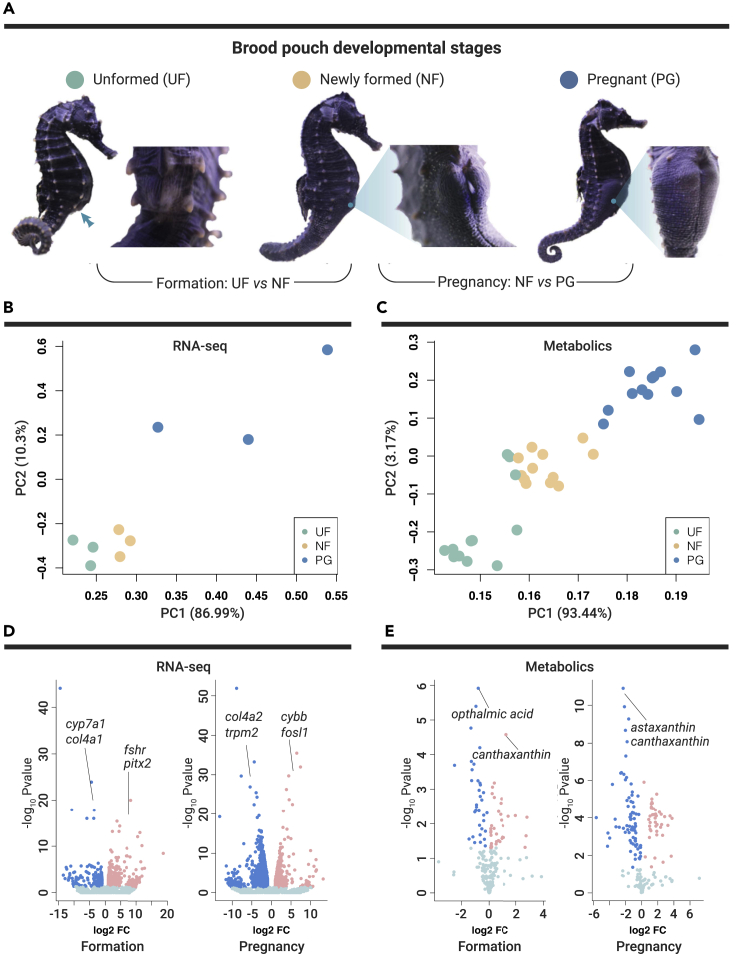
Transcriptome and Metabolome Mapping of Transcripts and Metabolites over Different Brood Pouch Developmental Stages in Seahorses (A) Illustration of unformed (UF), newly formed (NF), and pregnant (PG) brood pouches of seahorses. Two comparisons for the brood pouch formation and pregnancy are labeled. Principal-component analysis (PCA) for RNA-seq (B) and metabolome (C) data. Each point represents one biological replicate and points with different colors represent seahorses at different pouch developmental stages. Volcano plots showing the relative abundance of transcripts (D) and metabolites (E) in comparison for pouch formation and pregnancy. Important transcripts and metabolites are marked.

The results of two comparisons, that is, for pouch formation (unformed [UF] versus newly formed [NF]) and pregnancy (pregnant [PG] versus [NF]), of the lined seahorses (*H. erectus*) at UF, NF, and PG stages are shown in Figure 1A. In total, we quantified and annotated 21,232 transcripts and 210 metabolites (Tables S1–S4 and S13). Principal-component analysis (PCA) of both transcripts and metabolites showed large variations among seahorses at different stages of brood pouch development (Figures 1B and 1C). A total of 141 differentially expressed genes (DEGs) and 73 significantly different metabolites (SDMs) were identified in pouch formation analysis (Figure 1D; Tables S5 and S6), while 2,533 DEGs and 121 SDMs were identified in pregnancy comparison (Figure 1E; Tables S14 and S15).

**Figure 2:**
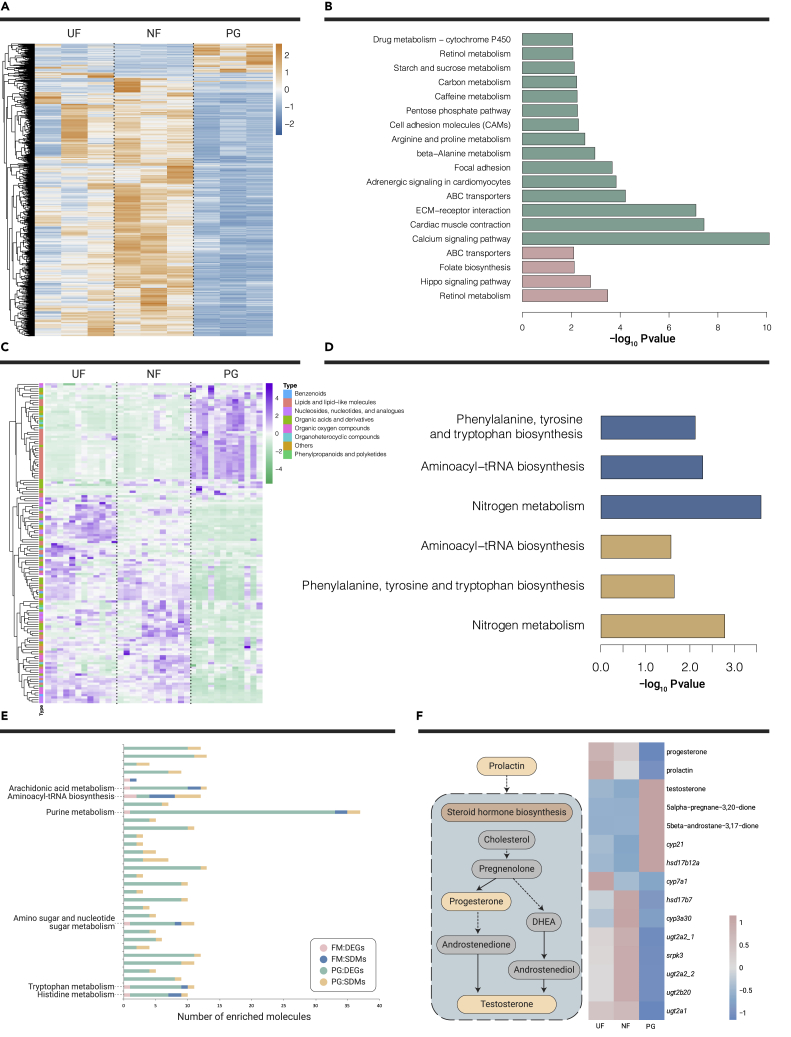
Expression Pattern and Enrichment Analysis of DEGs and SDMs over Different Pouch Developmental Stages (A–D) Heatmaps of DEGs (A) and SDMs (C). Significantly enriched pathways for DEGs (p < 0.01) (B) and SDMs (p < 0.05) (D). Pink and green bar charts in (B) represent pathways enriched for brood pouch formation and pregnancy processes, respectively; while the blue and orange bar charts in (D) indicate pathways enriched for SDMs identified for pouch formation and pregnancy processes, respectively. (E) Co-enriched pathways for both DEGs and SDMs. Co-enriched pathways for both brood pouch formation and pregnancy are labeled. (F) Changing patterns of DEGs and SDMs involved in steroid hormone metabolism. The left graph shows a simplified pathway of steroid hormone metabolism and the right graph shows changes in DEGs and SDMs participating in steroid hormone metabolism.

The expression levels of most DEGs were higher in seahorses in the NF stage and lower in the PG stage (Figure 2A). Two pathways (retinol metabolism and ABC transporters) were significantly enriched for both pouch formation and pregnancy (p < 0.01, Figure 2B; Table S7. Gene Ontology (GO) Enrichment Analysis of Differentially Expressed Genes (DEGs) Identified for the Brood Pouch Formation Process, Table S8. Gene Ontology (GO) Enrichment Analysis of Differentially Expressed Genes (DEGs) Identified for the Pregnancy Process, Table S9. Kyoto Encyclopedia of Genes and Genomes (KEGG) Enrichment Analysis of Differentially Expressed Genes (DEGs) Identified for the Brood Pouch Formation Process, Table S10. Kyoto Encyclopedia of Genes and Genomes (KEGG) Enrichment Analysis of Differentially Expressed Genes (DEGs) Identified for the Pregnancy Process). Meanwhile, SDMs exhibited diverse changes (Figure 2C). Specifically, aminoacyl-tRNA biosynthesis, nitrogen metabolism, as well as phenylalanine, tyrosine, and tryptophan biosynthesis were the three significantly enriched SDM pathways identified for both pouch formation and pregnancy (p < 0.05, Figure 2D; Tables S16 and S17). Of the 32 co-enriched pathways for transcriptome and metabolome analyses, 6 pathways, including aminoacyl-tRNA biosynthesis, were co-enriched for both pouch formation and pregnancy (Figure 2E; Tables S18 and S19). In particular, five SDMs and ten DEGs were mapped in the steroid hormone metabolism pathway (Figure 2F; Table S20). Upstream metabolites of the pathway (prolactin [PRL] and progesterone [PR]) were abundant in seahorses in the UF stage, while downstream metabolites of the pathways (testosterone [TE], 5-beta-androstane-3,17-dione and 5-alpha-pregnane-3,20-dione) were abundant in seahorses in the PG stage (Figure 2E). Unlike metabolic regulation, most DEGs participating in steroid hormone metabolism showed the highest expression levels in seahorses in the NF stage.

### Potential Multiple Functions of RA in the Development of the Brood Pouch

**Figure 3:**
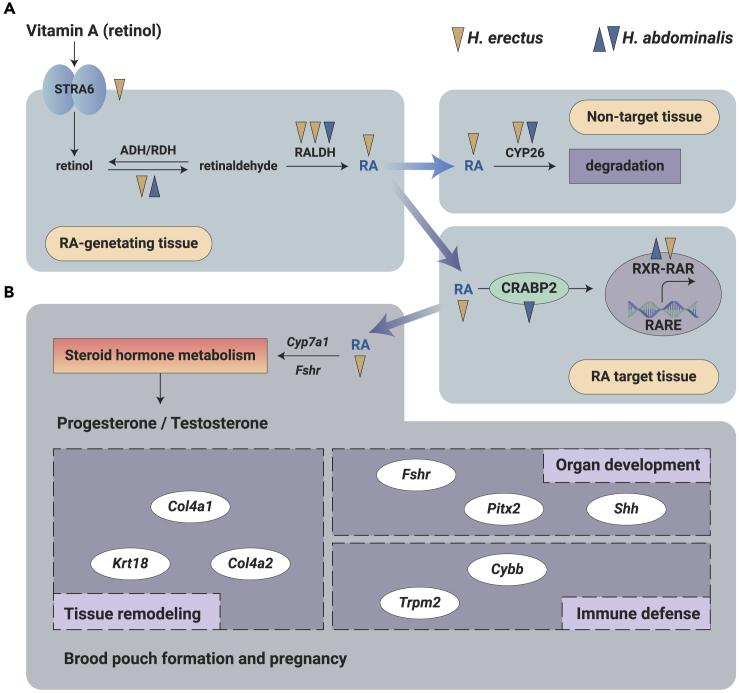
Role of RA in Seahorse Brood Pouch Formation and Pregnancy Detailed gene expression data are shown in Tables S11 and S12. (A) Role of RA synthesis and signaling during pregnancy of two seahorse species, *H. erectus* and *H. abdominalis*. RA synthesis and signaling pathway is simplified from Duester.^11^ Upward and downward arrows indicate upregulation and downregulation of genes and metabolites during pouch pregnancy, respectively. Each arrow represents one gene. (B) Summarized functions of RA target genes in seahorse brood pouch formation and pregnancy. RA might function upstream of testosterone and progesterone by regulating the expression of *fshr* and *cyp7a1*. Oval circles indicate RA target genes that were significantly differentially expressed during pouch formation or pregnancy and three rectangles indicate diverse functions that may be regulated by RA.

Retinol metabolism was significantly enriched in DEGs identified during both pouch formation and pregnancy. In fact, it was also the most significantly enriched pathway during pouch formation (Figure 2B; Tables S9 and S10). Expression level of six genes related to RA synthesis and signaling consistently decreased during pregnancy (Figure 3A; Table S11). In addition, 54 RA target genes were differentially expressed over different stages of pouch formation and pregnancy (Table S11). Expression of most RA target genes consistently decreased during pregnancy, while expression of several genes were increased in UF seahorses (e.g., cholesterol 7-alpha-monooxygenase [*cyp7a1*]) and PG (e.g., cytochrome b [558] subunit beta [*cybb*]) brood pouches (Table S11). These RA target genes participate in diverse interconnected functions, including steroid hormone metabolism (e.g., *cyp7a1* and follicle-stimulating hormone receptor [*fshr*]^19^), tissue remodeling (e.g., collagen alpha-1 (IV) chain [*col4a1*],^20^ collagen alpha-2 (IV) chain [*col4a2*],^21^ and keratin 18 [*krt18*]^22^), organ development (e.g., *fshr*^23^ and paired-like homeodomain 2 [*pitx2*]^24^), and immune defense (e.g., *cybb*^25^ and transient receptor potential cation channel subfamily M member 2 [*trpm2*]^26^) (Figure 3B). Intriguingly, five genes involved in RA synthesis and signaling, as well as 18 RA target genes, were also differentially expressed in *H. abdominalis* during pregnancy (Figure 3A; Table S12). Four RA target genes (*fshr*, *col4a2*, *col4a1*, and *krt18*) were differentially expressed during pregnancy in both *H. erectus* and *H. abdominalis* (Tables S11 and S12). In addition, data from a previous publication suggest that genes involved in RA signaling and synthesis tended to have higher expression levels in the pituitary and gonad, whereas RA target genes tended to have higher expression in the brood pouch (Figure S2).^11^

### Antioxidant Defenses Regulated by RA during Brood Pouch Formation and Pregnancy

**Figure 4:**
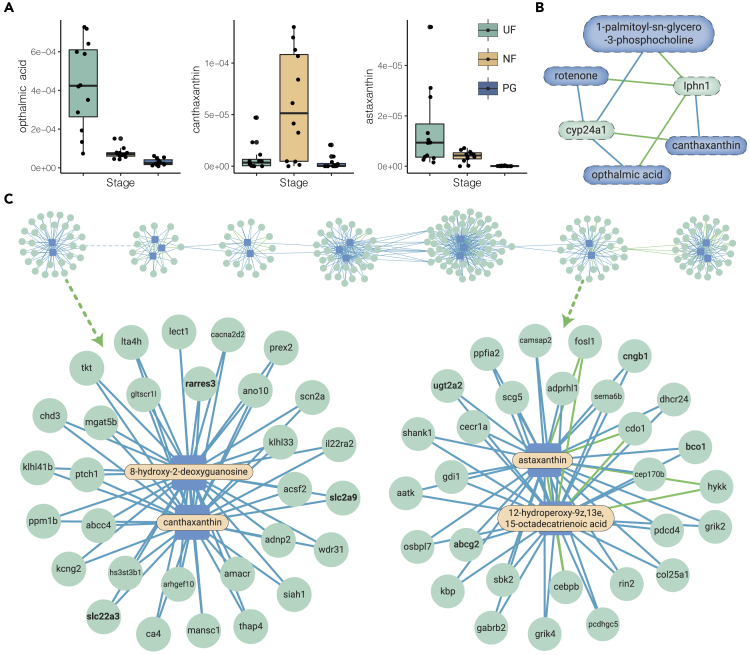
Interactional Network of Antioxidant Defense in Seahorse Brood Pouch Development (A) Boxplots showing the changing tendency of one oxidant and two antioxidants over different pouch developmental stages in seahorses. Each point represents one replicate (n = 12). Center line, median; box limits, upper and lower quartiles; whiskers, 1.5× the interquartile range. (B and C) Significant correlations between significantly different antioxidants and DEGs (correlation coefficient >0.9 and p < 0.01). Fonts of antioxidants and genes involved in transporting or related to RA are in bold. Metabolites and genes are indicated with blue and green background colors, respectively. Blue and green lines indicate significant positive and negative correlations, respectively. (B) Correlations between canthaxanthin and ophthalmic acid with other DEGs identified for brood pouch formation. (C) Correlations between canthaxanthin (left) and astaxanthin (right) with other DEGs identified during pregnancy.

Canthaxanthin, acting as an antioxidant,^27^ showed the highest fold change (7-fold) in seahorses in the NF stage compared with the UF stage, whereas ophthalmic acid, an oxidant,^28^ showed the greatest (5.9-fold) change in seahorses in the UF stage compared with the NF stage (Figure 4A). Compared with seahorses in the NF stage, pregnant seahorses had lower levels of antioxidants (canthaxanthin, −19.1-fold; astaxanthin, −50.3-fold) (Figure 4A). Expression of glutathione *S*-transferase (GST) also significantly decreased in pregnant seahorses (Table S6).

Ophthalmic acid and canthaxanthin showed reverse correlations with the same DEGs identified during pouch formation (Figure 4B; Table S21). During pregnancy, both canthaxanthin and astaxanthin were core metabolites of the regulatory network and showed a positive correlation with many genes, particularly those participating in transportation, including ATP-binding cassette superfamily G member 2 (*abcg2*) (Figure 4C; Table S22). Meanwhile, canthaxanthin showed a positive correlation with RA receptor responder protein 3 (*rarres3*), and astaxanthin showed a positive correlation with UDP glucuronosyltransferase 2 family, polypeptide A2 (*ugt2a2*) and beta,beta-carotene 15,15′-dioxygenase (*bco1*) during pregnancy (Figure 4C). *Rarres3*, *ugt2a2*, and *bco1* were all involved in RA metabolism and signaling (Tables S10 and S11).

## Discussion

In this study, we quantified transcripts and metabolites over different seahorse brood pouch developmental stages and suggested multiple roles of RA in pouch formation and pregnancy by regulating steroid hormone metabolism and antioxidant defense. In seahorses, interruption of PRL synthesis leads to disruption of brooding tissues and spontaneous abortions during pregnancy as PRL production is essential for PR and TE secretion.^4^ However, no study has examined changes in sex steroid hormones over different stages of brood pouch development or how they affect pouch development in seahorses. Here, we detected high levels of PRL and PR in seahorses before pregnancy and high levels of TE during pregnancy (Figure 2F). Furthermore, we indicated the different roles of upstream and downstream metabolites of steroid hormone metabolism in seahorse brood pouch formation and pregnancy (Figure 2F). In addition, we observed nonsynchronous regulation at the transcriptional and metabolic levels (Figure 2F). Owing to their diverse roles, the concentrations of sex steroids fluctuate during the breeding cycle of the lined seahorses.^29^ Future research on specific sex hormones may help to better illustrate the complex roles of steroid hormone metabolism in brood pouch development.

In addition to the roles of steroid hormones, our results may indicate that RA regulates 54 downstream target genes with diverse functions, indicating its potential roles in seahorse brood pouch development (Figure 3). In fact, involvement of RA in teleost development and regeneration has been widely reported,^13^^,^^30^^,^^31^ and patterned expression of RA-related genes may play specific roles (e.g., tissue remodeling, organ development, and immune defense) in seahorse brood pouch development.^9^ For example, PITX2, a transcription factor known for its role in left-right symmetry,^24^^,^^32^ may control seahorse brood pouch symmetry by regulating the Wnt signaling and collagen-related genes.^33^ Meanwhile, CYBB, a super-oxide-generating enzyme conserved in most eukaryotic groups, is involved in antioxidant immune defense as it is activated in response to pathogens.^25^ Diverse and pleiotropic functions of genes in the brood pouch may be responsible for their diverse expression pattern (Table S11).

Contradictory results have been reported regarding the relationship between the RA signaling pathway and steroid hormone metabolism. On the one hand, conditional deletion of aldehyde dehydrogenase 1 family member A2 (*aldh1a2*), the enzyme that metabolizes the vitamin A-intermediate retinaldehyde into RA, results in embryonic pituitary dysmorphology and alters hormone expression.^34^ On the other hand, TE has been reported as the key upstream signal that controls RA biogenesis by promoting the expression of genes encoding RA synthesizing enzymes.^35^ Our results indicate that RA may function upstream of steroid hormone metabolism in seahorses as demonstrated by the consistent decrease in levels of upstream molecules of steroid hormone metabolism with RA during pregnancy (Figures 2F and 3A). Therefore, PRL, PR, and TE play important roles in the maintenance of brood pouch structure and function of brood pouch.^4^^,^^36^ Whether RA regulates pouch development by steroid hormones requires further investigation.

DEGs that participate in RA signaling and pathway, or that are regulated by RA, were also identified in *H. abdominalis*, suggesting a conserved role of RA in *Hippocampus* brood pouch development (Figure 3A; Tables S11 and S12). Of the four RA target genes identified in comparison of the pregnancy process in both *H. erectus* and *H. abdominalis*, *fshr* was reportedly expressed during the luteal phase in the secretory endometrium of the mammalian uterus^23^ and may be involved in the formation of placenta-like tissue during pregnancy.^37^ FSHR may also be involved in pouch formation by affecting TE production.^19^ Meanwhile, COL4A1 and COL4A2 are the major structural components of basement membranes^20^^,^^21^ and KRT18 is a member of the intermediate filament family of cytoskeletal proteins;^22^ all three of which may be involved in brood pouch tissue remodeling. Our results may indicate the various roles of RA in seahorse brood pouch formation and pregnancy by regulating downstream genes with diverse functions. In addition, different tissue-specific expression pattern of genes participating in RA signaling and synthesis (Figure 3A) and RA target genes (Figure 3B) may indicate that changes in RA level was resulted from the differential expression of genes in the pituitary and gonad, which further regulated pouch development by regulating the expression of RA target genes in the brood pouch (Figure S2). Meanwhile, in mammals, RA is required to promote differentiation of the mesenchyme into the future uterus and vagina by acting on the Mullerian duct, and plays a vital role in morphological and functional differentiation of female reproductive organs.^38^^,^^39^ Therefore, future studies comparing the specific morphological and functional changes in the reproductive organs of female mammals and male seahorses during pregnancy post-RA challenge may better illustrate the role of RA in male and female pregnancy.

This study serves as the first application of metabolome in seahorse for identifying important pathways and metabolites possibly related to brood pouch formation and pregnancy. As the co-enriched pathways of DEGs and SDMs identified in both pouch formation and pregnancy, aminoacyl-tRNA biosynthesis may participate in the formation of placenta-like tissues during male pregnancy by facilitating the growth of collagenous fibers.^40^ Furthermore, antioxidant defense regulation, which is necessary to maintain a stable low concentration of reactive oxygen species,^41^ plays a key role in male pregnancy (Figure 4). Reproduction is an energetically demanding activity, and the metabolic rate of pregnant seahorses increases from 10% to 52% over pre-gravid levels.^42^ The increasing metabolic rate could induce oxidative stress during pregnancy by consuming antioxidants and enzymes (Figure 4A), which may weaken the immune defense^43^ and facilitate successful gestation. The trade-off between immunological tolerance and embryo rejection accompanies the evolution of unique male pregnancy.^44^ Accordingly, the increased susceptibility to oxidative stress in seahorses during pregnancy might result from the necessity to strike a balance between reproduction and survival that contributes to seahorse reproduction.^45^^,^^46^

In addition, the protective role of transporters against oxidative stress may be responsible for their positive correlation with antioxidants (Figure 4C). For example, ABCG2, an ABC transporter that discharges a variety of toxic substances, protects cells from reactive oxygen species-mediated cell damage.^47^ We also detected a positive correlation between astaxanthin and genes participating in RA signaling (Figure 4C). The consistent decreasing trend in RA expression and antioxidants, as well as their correlation during pregnancy, may suggest that antioxidant defense during brood pouch development is partly under the control of RA. Low RA levels in the cell milieu are relevant to the expression of several antioxidants and antioxidant enzymes, including GST;^18^ in mammals, a higher oxidative state caused by RA treatment promotes secretion of vascular endothelial growth factor, which may be necessary for successful pregnancy.^48^ Therefore, decreasing RA and antioxidants in the seahorse during pregnancy indicates that an RA-related antioxidant defense mechanism plays a key role in successful reproduction during seahorse pregnancy, which is similar to that in mammals. Correlations between antioxidants and genes with different functions could indicate the synergistic network of molecules involved in brood pouch development and require further research.

Certain limitations were noted in this study. First, is the potential bias associated with the sampling design for transcriptome analysis. This may result in allometric changes in organ properties due to the samples being collected from animals in different stages. However, quantifying and extracting RNA from half a testis or the pituitary gland from one seahorse is challenging due to their light weight. Although this bias likely had minimal effects on comparison of the pouch pregnancy process, since both NF and PG seahorses contain mature tissues, it is important to consider these issues when interpreting transcriptome data for the pouch formation process. In addition, similar to what has been reported in previous seahorse studies,^9^^,^^49^ we used three replicates per group for transcriptomic analysis and chose edgeR to identify DEGs due to its power in dealing with a low number of replicates.^50^ Despite the reasonable DEG dataset we obtained, Schurch et al.^50^ stated that only a tiny fraction of DEGs can be detected with three replicates per group. Hence, future transcriptome studies with a higher number of replicates will enrich the gene dataset responsible for pouch development. Furthermore, although we carefully interpreted the correlation networks, we did not focus on the specific correlations between a single transcript and metabolite due to the low sampling size (n = 6) used for Pearson correlation analysis. Hence, although an increased sample size may serve to generate more convincing correlation results, we believe that the reasonable positive correlations presented here between antioxidants and RA-related genes and transporters during pregnancy will not be significantly impacted.

### Conclusion

We showed the general trends in transcript and metabolite changes over different stages of seahorse brood pouch development and highlighted the potential roles of RA, and its target genes, in brood pouch formation and pregnancy. This first application of metabolome analysis in the lined seahorse uncovered an increased susceptibility to oxidative stress in seahorses during pregnancy, which may have resulted from decreased RA expressions. We also identified certain molecules with important functions in seahorse brood pouch formation and pregnancy, which will promote future specific functional brood pouch studies post-RA and stress challenge in syngnathids.

## Material and Method

### Ethical Approval

All the experiments were carried out with an approval from the Experimental Animal Ethics Committee of the South China Sea Institute of Oceanology, Chinese Academy of Sciences, China.

### Experimental Materials

A total of 100 lined seahorses were obtained from the Shenzhen Seahorse Breeding Center (Shenzhen, China) in August 2017. These seahorses were categorized into three developmental stages according to brood pouch morphology: (1) unformed (UF) stage comprising 50 seahorses (5.0–5.7 cm in length and 0.39–0.54 g in weight) with unformed brood pouches; (2) newly formed (NF) stage, which included 25 male seahorses (6.2–7.4 cm in length and 0.78–1.43 g in weight) with newly formed brood pouches; and (3) pregnant (PG) stage, including 25 male seahorses (9.0–9.3 cm in length and 2.42–3.55 g in weight) during pregnancy.

### Tissue Sampling

The seahorse dissection and sampling processes were performed on ice. After dissection, UF-stage seahorse gonads were observed under a microscope, and only male seahorses with testes were retained. Embryos were gently removed from pregnant seahorses before sampling. A total of 18 individual seahorses in each of the three stages were used. Muscles from six seahorses from each stage were used to detect RA, PRL, PR, and TE; another 12 seahorses in each stage were used for transcriptome and metabolome analyses. From each animal, half of the pituitary gland, testis, and brood pouch tissue were dissected, pooled, and quickly frozen in liquid nitrogen until transcriptome analysis. For the UF stage, skin tissue was collected from the primordial brood pouch area and designated as the pouch sample. After transcriptome sampling, all remaining tissues from each animal were then frozen separately in liquid nitrogen for metabolome analysis. Finally, 12 samples from each stage were used for metabolome analysis and 3 out of the 12 samples were randomly selected and used for transcriptome analysis^9^^,^^49^ (Figure S1).

### RNA Sequencing and Detection and Analysis of DEGs

RNA (three replicates per stage) was extracted using TRIzol (Invitrogen, USA) and RNA quality was assessed using an Agilent 2100 Bioanalyzer (Agilent Technologies, Santa Clara, CA, USA). Each replicate was used to generate an independent library. The RNA sequencing paired-end (PE) libraries were prepared using a NEBNext Ultra RNA Library Prep Kit for Illumina (NEB, Ipswich, MA, USA), following the manufacturer's protocols. Each library was sequenced on an Illumina HiSeq platform (Illumina, San Diego, CA, USA) with 150 bp PE reads. A total of approximately 489 million clean PE reads were generated. After reads filtering and removal of rRNA-mapped reads,^51^ high-quality reads were mapped to the reference genome of the lined seahorse (NCBI accession number: PRJNA347499)^52^ and gene abundance was quantified using the FPKM (fragments per million mapped reads) method.^53^ Based on the expression pattern of all genes, PCA was performed using R package gmodels (http://www.r-project.org/) for sample relationship analysis. DEGs were detected with the edgeR package using the following criteria: >2-fold change >2 and false discovery rate <0.05.^50^^,^^54^ Using the annotations of all identified transcripts as a reference, gene ontology (GO) and Kyoto Encyclopedia of Genes and Genomes (KEGG) enrichment analysis were conducted using DAVID v.6.8 (https://david.ncifcrf.gov/) and KOBAS software,^55^ respectively. GO terms or KEGG pathways with a calculated p value < 0.01 were considered significantly enriched.

### Detection of DEGs Related to RA in Seahorse

We first recorded RA-related genes identified in previous studies, including genes participating in the RA synthesis and signaling pathway,^11^ as well as those with expression regulated by RA (RA target genes).^56^ We then searched for RA-related genes from the DEGs identified by comparing both the pouch formation and pregnancy process of *H. erectus*. To better illustrate the role of RA in *Hippocampus*, we also searched RA-related genes from among the DEGs identified in the pregnancy process of the pot-bellied seahorse *Hippocampus abdominalis*.^9^ We then used in-house R scripts to identify RA-related DEGs associated with the pregnancy process of both species and investigated their expression tendency. To reduce errors caused by inconsistency in gene names from different studies, only gene symbols generated by the Ensembl dataset were used when gene lists identified from different studies were compared.

### Metabolite Extraction, Detection, and Analysis

All remaining tissues from the 36 seahorses (12 replicates per stage) were freeze-dried separately (Boyikang, Beijing, China) and made into powder using a tissue crushing apparatus (Guangzhou Good, Guangzhou, China). After mixing evenly, approximately 20 mg of tissue per sample was collected for UHPLC-QE Orbitrap/MS analysis according to the standard protocol (Biotree, Shanghai, China). The resulting data matrix, including the peak number, sample name, and normalized peak area was imported to the SIMCA 14.1 software package (v.14.1, Umea, Sweden) for PCA. Metabolites with variable importance for the projection values > 1 and p value < 0.05 were identified as SDMs. Commercial databases, including KEGG (http://www.genome.jp/kegg/) and MetaboAnalyst (http://www.metaboanalyst.ca/) were utilized to search for metabolite pathways. Pathways with a calculated p value < 0.05 were considered significantly enriched.

### Detection of RA, PRL, PR, and TE

As a complement to the non-target metabolome, using enzyme-linked immunosorbent assay (ELISA) we detected levels of PRL, PR, and TE due to their reported roles in seahorse brood pouch development.^4^ RA was also detected using the same method. There were six replicates per developmental stage. Muscle tissues were used for RA, PRL, PR, and TE detection. Muscles in each sample were maintained at 2°C–8°C after melting and were homogenized by grinding in PBS solution (pH 7.4). These samples were centrifuged at 3,000 rpm for 20 min and the supernatant was collected and reserved for detection. RA (cat. no. RJ-27871), PRL (cat. no. RJ-21563), TE (cat. no. RJ-21574), and PR (cat. no. RJ-21719) were all detected according to the ELISA manufacturer's instructions (Renjie, Shanghai, China). In brief, the purified antibody was used to coat microtiter plate wells and produce a solid-phase antibody. The muscle supernatant was then added to the wells and combined with a horseradish peroxidase (HRP)-labeled antibody to form an antibody-antigen-enzyme-antibody complex. 3,3′,5,5′-Tetramethylbenzidine (TMB) was added for coloration after washing thoroughly; TMB turned blue after HRP catalysis and yellow after the addition of sulfuric acid solution. Absorbance (optical density) was measured at 450 nm using a microplate reader (Synergy H1, BioTek, USA), and the concentration of the target protein in the sample was determined using the standard curve method.

### Integrative Analysis of Metabolome and Transcriptome

For each of the two processes (pouch formation and pregnancy), two separate KEGG analyses were conducted for identified DEGs and SDMs. We then compared the enriched KEGG pathways of the DEGs and SDMs identified for the same process to identify the co-enriched pathways of transcriptome and metabolome analysis. Pearson correlation coefficients between DEGs and SDMs identified for comparison of the same pouch process were further calculated. DEGs and SDMs with a correlation coefficient >0.9 and p value < 0.01 were imported to Cytoscape (v.3.3.0),^57^ which visualized and clarified the metabolomic and transcriptome data and built networks between genes and metabolites. Here, we focused on the entire correlation network between the transcriptome and metabolome, rather than one specific correlation between a single transcript and metabolite. Moreover, according to the results published previously,^58^^,^^59^ we did not further correct the p values.

## Lead Contact Website

http://sourcedb.scsio.cas.cn/cn/rck/jcqn/202004/t20200410_5536905.html.

## Web Resources

Sequencing raw data of transcriptome were deposited at NCBI under the accession number PRJNA628893.

## Supplemental Information

Document S1. Figures S1–S4 and Tables S1–S3, S11, S12, S18, and S20

Table S4. Gene Abundance Shown by Fragments per Million Mapped Reads (FPKM)

Table S5. Differentially Expressed Genes (DEGs) Identified for the Brood Pouch Formation Process

Table S6. Differentially Expressed Genes (DEGs) Identified for the Pregnant Process of the Brood Pouch

Table S7. Gene Ontology (GO) Enrichment Analysis of Differentially Expressed Genes (DEGs) Identified for the Brood Pouch Formation Process

Table S8. Gene Ontology (GO) Enrichment Analysis of Differentially Expressed Genes (DEGs) Identified for the Pregnancy Process

Table S9. Kyoto Encyclopedia of Genes and Genomes (KEGG) Enrichment Analysis of Differentially Expressed Genes (DEGs) Identified for the Brood Pouch Formation Process

Table S10. Kyoto Encyclopedia of Genes and Genomes (KEGG) Enrichment Analysis of Differentially Expressed Genes (DEGs) Identified for the Pregnancy Process

Table S13. Relative Quantity of Annotated Metabolites across Different Brood Pouch Developmental Stages

Table S14. Significant Different Metabolites (SDMs) Identified for the Brood Pouch Formation Process

Table S15. Significantly Different Metabolites (SDMs) Identified for the Pregnancy Process

Table S16. Kyoto Encyclopedia of Genes and Genomes (KEGG) Enrichment Analysis of Significantly Different Metabolites (SDMs) Identified for the Brood Pouch Formation Process

Table S17. Kyoto Encyclopedia of Genes and Genomes (KEGG) Enrichment Analysis of Significantly Different Metabolites (SDMs) Identified for the Pregnancy Process

Table S19. Co-enriched Pathways of Both Transcriptomics and Metabolomics for the Pregnancy Process

Table S21. Correlation Analysis between Differentially Expressed Genes (DEGs) and Significantly Different Metabolites (SDMs) Identified for the Brood Pouch Formation Process

Table S22. Correlation Analysis between Differentially Expressed Genes (DEGs) and Significantly Different Metabolites (SDMs) Identified for Pregnancy Process

Document S2. Article plus Supplemental Information
