# Supplementary material for: Regulatory Role of Retinoic Acid in Male Pregnancy of the Seahorse
Source: Innovation (Camb). 2020 Oct 13;1(3):100052. doi: 10.1016/j.xinn.2020.100052 (PMC8454549; doi:10.1016/j.xinn.2020.100052)
Supplement: Document S1. Figures S1–S4 and Tables S1–S3, S11, S12, S18, and S20 [file mmc1.pdf]

XINN, Volume *1*

Supplemental Information

Regulatory Role of Retinoic Acid in Male Pregnancy of the Seahorse

Chunyan Li, Yongxin Li, Geng Qin, Zelin Chen, Meng Qu, Bo Zhang, Xue Han, Xin Wang, Pei-yuan Qian, and Qiang Lin

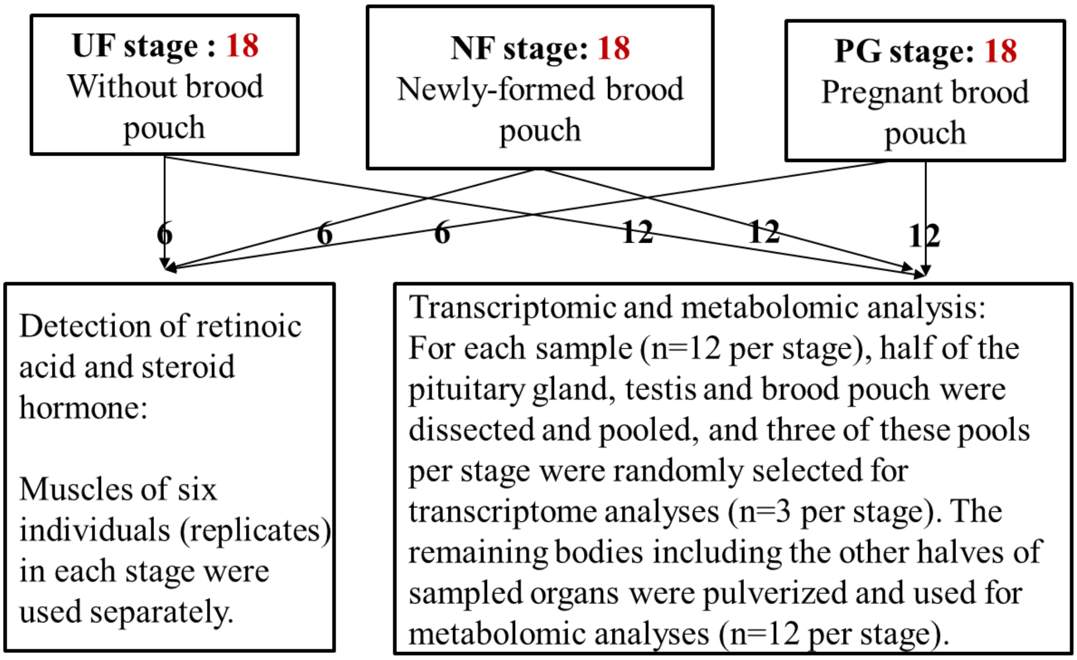

Figure S1. Illustration of seahorse sampling and using in this study. Digits in red font indicate total sampling number in each pouch developmental stage and digits on the arrows indicate the sampling number in each stage for different analyses.

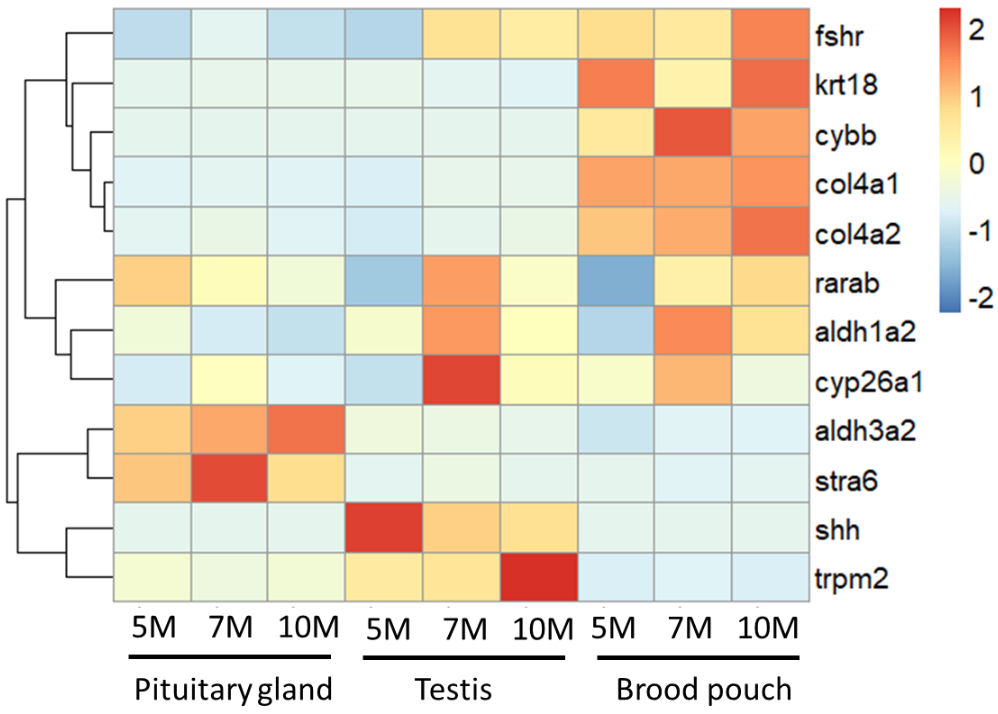

Figure S2. Heatmap of the spatio-temporal specific expression pattern of the lined seahorse (raw data downloaded from NCBI: SRA392578)[10]. The x-axis represents different tissues from seahorses at different developmental stages. 5M, 7M and 10M represent 5, 7, and 10-month old male seahorses, respectively. The y-axis stands for RA-related genes.

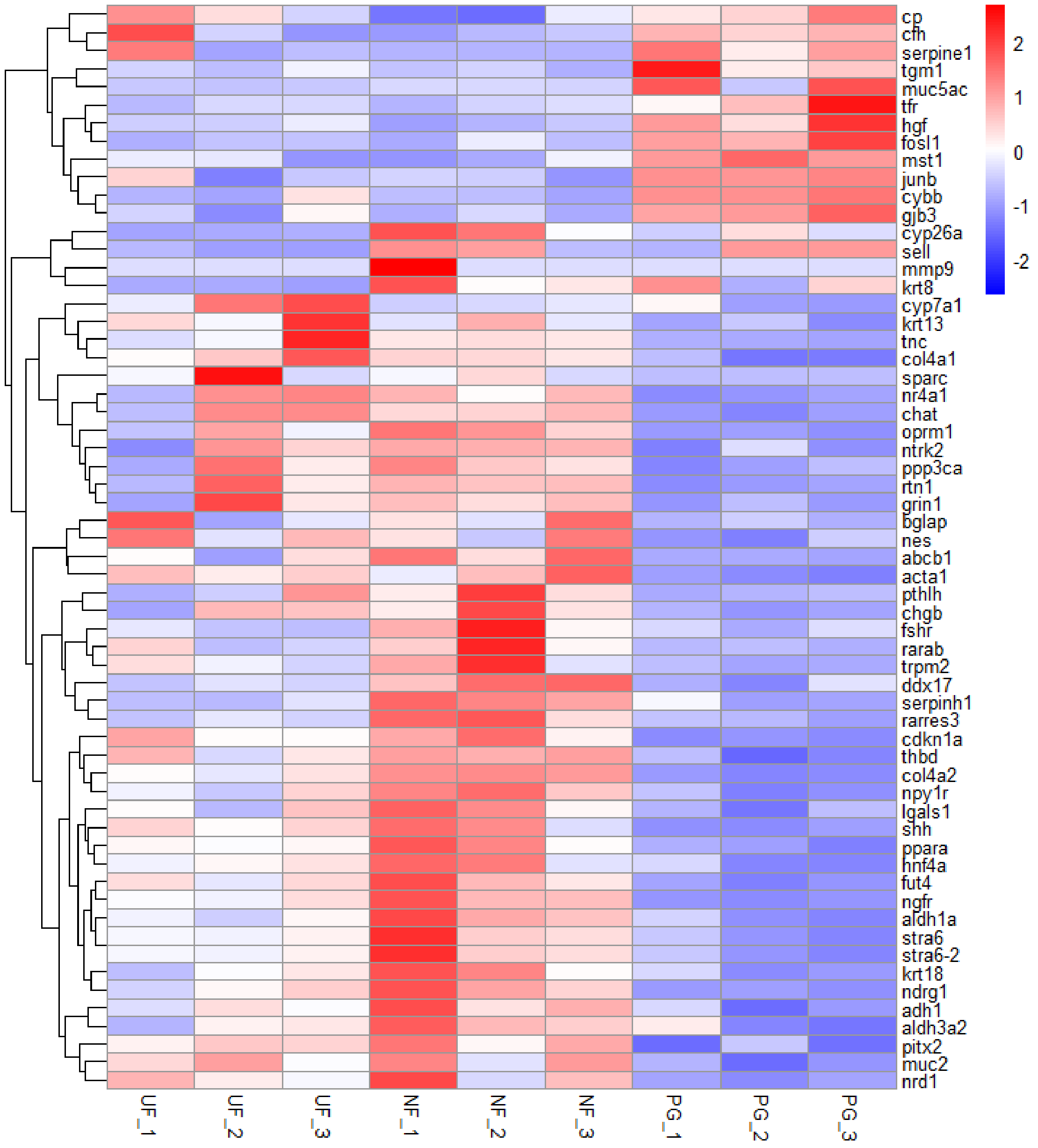

Figure S3. Heatmap of the expression tendency of RA-related genes across different brood pouch developmental stages. The x-axis represents samples at different pouch developmental stages and the y-axis represents RA-related genes.

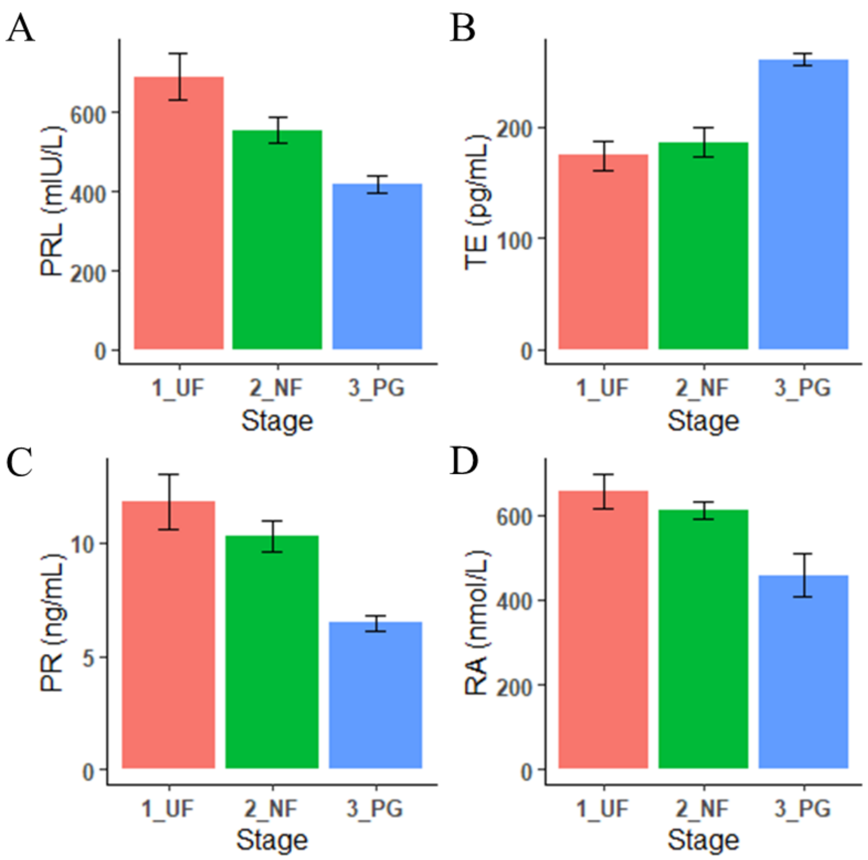

Figure S4. Histogram of the changing tendency of metabolites across different brood pouch developmental stages. (A) Prolactin (PRL). (B) Testosterone (TE). (C) Progesterone (PR). (D) Retinoic acid (RA). UF, NF and PG represent stage with unformed brood pouch, newly formed brood pouch, and brood pouch during pregnancy, respectively. Each stage has 6 biological replicates.

Table S1. Statistics of RNA-seq data.

Sample	Before Filter						After Filter					
	Clean Reads	Clean Data(bp)	Q20(%)	Q30(%)	N(%)	GC(%)	HQ Clean Reads	HQ Clean Data(bp)	Q20(%)	Q30(%)	N(%)	GC(%)
UF-1	55433344	8315001600	8185645732 (98.44%)	7982555284 (96.00%)	1007229 (0.01%)	4461782653 (53.66%)	54631848	8108123310	8030320718 (99.04%)	7863624037 (96.98%)	918014 (0.01%)	4350122685 (53.65%)
UF-2	56099652	8414947800	8282624545 (98.43%)	8074368697 (95.95%)	1017741 (0.01%)	4531333984 (53.85%)	55276180	8205958945	8126175495 (99.03%)	7955076068 (96.94%)	928619 (0.01%)	4418033431 (53.84%)
UF-3	51482962	7722444300	7603630977 (98.46%)	7416734146 (96.04%)	938666 (0.01%)	4097286390 (53.06%)	50769914	7539172585	7467270036 (99.05%)	7313460225 (97.01%)	854167 (0.01%)	3999085910 (53.04%)
NF-1	54168874	8125331100	8003890019 (98.51%)	7811967916 (96.14%)	980087 (0.01%)	4367090967 (53.75%)	53420786	7931695968	7858394973 (99.08%)	7700343915 (97.08%)	898286 (0.01%)	4262219397 (53.74%)
NF-2	50104228	7515634200	7401672183 (98.48%)	7219849226 (96.06%)	912685 (0.01%)	4111872365 (54.71%)	49415238	7334885200	7265645680 (99.06%)	7115247679 (97.01%)	832591 (0.01%)	4012768481 (54.71%)
NF-3	63606320	9540948000	9397910315 (98.50%)	9168607511 (96.10%)	1153287 (0.01%)	5163922475 (54.12%)	62750650	9317456714	9229686531 (99.06%)	9039451025 (97.02%)	1051173 (0.01%)	5042249180 (54.12%)
PG-1	50593674	7589051100	7476539724 (98.52%)	7297662377 (96.16%)	921982 (0.01%)	4047183238 (53.33%)	49884558	7406457008	7337960483 (99.08%)	7190051574 (97.08%)	840100 (0.01%)	3949079228 (53.32%)
PG-2	54550622	8182593300	8059733175 (98.50%)	7859634143 (96.05%)	988112 (0.01%)	4495487557 (54.94%)	53757744	7984664108	7907972478 (99.04%)	7740980926 (96.95%)	901618 (0.01%)	4386408360 (54.94%)
PG-3	52753606	7913040900	7803846435 (98.62%)	7625778800 (96.37%)	962820 (0.01%)	4253833323 (53.76%)	52054896	7733211233	7665280678 (99.12%)	7516496768 (97.20%)	877281 (0.01%)	4156630698 (53.75%)

Table S2. Reads mapping result of RNA-seq data.

Sample	Mapped to rRNA Database		Mapped to the genome of *H. erectus*		
	HQ Clean Reads	Unmapped Reads	Unique Mapped Reads	Multiple Mapped reads	Mapping Ratio
UF-1	54631848	54606142 (99.95%)	43533947 (79.72%)	3735956 (6.84%)	0.8657
UF-2	55276180	55253848 (99.96%)	44965824 (81.38%)	3697338 (6.69%)	0.8807
UF-3	50769914	50735808 (99.93%)	40973761 (80.76%)	2915644 (5.75%)	0.8651
NF-1	53420786	53397646 (99.96%)	43047014 (80.62%)	3228906 (6.05%)	0.8666
NF-2	49415238	49401256 (99.97%)	40713409 (82.41%)	2944380 (5.96%)	0.8837
NF-3	62750650	62727472 (99.96%)	51050593 (81.38%)	4091784 (6.52%)	0.8791
PG-1	49884558	49872392 (99.98%)	40594041 (81.40%)	2989508 (5.99%)	0.8739
PG-2	53757744	53751606 (99.99%)	42278993 (78.66%)	4502458 (8.38%)	0.8703
PG-3	52054896	52044182 (99.98%)	41780335 (80.28%)	3752182 (7.21%)	0.8749

Table S3. Annotation of transcripts identified from RNA-seq data.

Sample Name	Known Gene Number		New Gene Number		All Gene Number	
UF-1	15673 (75.39%)		3016		18689	
UF-2	16019 (77.06%)	16966 (81.61%)	3076	3246	19095	20212
UF-3	15934 (76.65%)		3071		19005	
NF-1	16283 (78.33%)		3158		19441	
NF-2	16164 (77.76%)	17233 (82.90%)	3115	3289	19279	20522
NF-3	16266 (78.25%)		3130		19396	
PG-1	15607 (75.08%)		2964		18571	
PG-2	15622 (75.15%)	16812 (80.87%)	2945	3177	18567	19989
PG-3	15842 (76.21%)		3027		18869	

Table S4. Gene abundance showed by Fragments per million mapped reads (FPKM) (Excel Table).

Table S5. Differentially expressed genes (DEGs) identified for the brood pouch formation process (Excel Table).

Table S6. Differentially expressed genes (DEGs) identified for the pregnant process of brood pouch (Excel Table).

Table S7. Gene ontology (GO) enrichment analysis of differentially expressed genes (DEGs) identified for the brood pouch formation process (Excel Table).

Table S8. Gene ontology (GO) enrichment analysis of differentially expressed genes (DEGs) identified for the pregnancy process (Excel Table).

Table S9. Kyoto encyclopedia of genes and genomes (KEGG) enrichment analysis of differentially expressed genes (DEGs) identified for the brood pouch formation process (Excel Table).

Table S10. Kyoto encyclopedia of genes and genomes (KEGG) enrichment analysis of differentially expressed genes (DEGs) identified for the pregnancy process (Excel Table).

Table S11. Expression pattern of differentially expressed genes (DEGs) relevant to retinoic acid synthesis and signaling in *H. erectus*.

Gene Symbol	UF-1	UF-2	UF-3	NF-1	NF-2	NF-3	PG-1	PG-2	PG-3
adh1	84.65	117.28	99	188.98	115.31	138.59	81	26.91	50.56
aldh1a	57.58	45.86	65.34	124.46	90.35	82.41	46.1	24.61	19.94
cyp26a	1.04	1.37	2.09	27.44	24.15	9.83	5.16	13.51	6.64
aldh3a2	8.7	18.69	19.47	35.13	24.98	22.84	18.92	3.79	2.11
stra6	17.38	16.47	20.35	44.9	24.56	22.73	11.35	5.08	3
rarab	0.85	0.24	0.34	0.89	1.85	0.63	0.21	0.23	0.15
fut4	10.84	7.79	11.29	19.27	12.93	10.37	3.74	1.81	2.9
tgm1	3.28	2.76	4.1	2.9	3.23	2.4	10.48	4.86	5.9
bglap	37.91	0.76	9.97	17.74	9.74	34.76	2.5	6.12	2.13
cyp7a1	39.06	90.88	105.45	27.67	32.44	36.93	46.79	12.99	11.91
pitx2	2.16	2.59	2.52	3.51	2.1	2.98	0.37	1.43	0.43
fshr	11.35	7.49	7.11	21.86	37.11	14.49	9.76	4.82	10.22
sparc	0.31	1.78	0.15	0.32	0.6	0.15	0	0	0
mmp9	0	0	0	1.95	0	0	0	0	0
hgf	7.46	7.56	9.3	5.14	6.31	7.31	15.87	12.03	21.28
ppara	14.86	14.16	14.8	25.95	22.68	14.59	9.08	8.13	5.8
junb	26.03	9.07	16.51	17.54	16.59	11.03	32.21	32.07	33.26
tnc	1.12	1.52	4.96	1.97	2.18	2.03	0.43	0.33	0.27
pthlh	8.43	10.64	21.29	15.26	27.63	16.37	7.87	8.68	9.53
shh	4.64	3.78	4.66	7.08	6.36	2.85	1.16	1.02	1.54
hnf4a	25.72	28.82	30.99	48.16	45.42	23.89	22.01	11.37	11.05
ngfr	4.9	4.62	6.12	11.08	7.43	7.29	1.34	0.99	1.4
col4a1	7.11	8.84	12.87	8.58	8.42	7.94	4.8	2.35	2.56
col4a2	4.14	3.5	4.76	6.96	7.14	6.74	1.42	0.88	0.98
krt13	60.22	46.14	109.16	40.73	72.07	42.51	22.05	32.92	15.61
krt8	49.28	48.92	42.01	202.41	100.03	112.3	166.28	51.96	125.23
thbd	12.6	7.71	10.51	13.56	12.91	13.44	6.73	3.04	4.43
serpinh1	4.97	4.8	6.34	13.1	11.91	10.67	7.15	3.84	4.02
muc2	19.39	23.84	15.93	25.97	13.75	24	10.27	4.57	7.58
cfh	1290.73	471.41	233.17	247.99	371.93	430.77	911.22	799.01	902.48
serpine1	23.06	4.66	6.49	5.6	5.74	5.72	23.74	13.7	20.14
muc5ac	0.15	0	0.11	1.37	1.12	0.94	14.71	0.28	14.99
cybb	22.04	17.98	47.17	24.64	23.95	17.86	68.28	67.48	74.41
abcb1	4.72	2.14	5.59	8.25	5.57	8.68	2.49	2.46	2.29
oprm1	0.69	2.06	1.09	2.45	2.18	1.63	0.29	0.32	0.2
rtn1	8.87	23.76	14.74	18.5	17.17	17.7	5.79	6.67	7.54
nr4a1	1.32	3.27	3.39	2.86	2.12	2.85	0.78	0.92	1.08
chat	0.42	1.28	1.28	0.91	0.92	1.05	0.24	0.14	0.26
ntrk2	1.85	6.45	5.2	6.03	6	5.81	1.61	3.52	1.99
nes	4.78	2.48	3.84	3.28	2.12	4.69	1.42	1.12	2.24
krt18	134.49	205.12	235.39	400.71	341.68	210.25	163.74	73.98	92.95
ndrg1	276.54	393.75	489.9	773.48	583.37	478.2	151.13	157.03	127.83
ddx17	17.83	21.31	19.18	30.86	40.76	41.7	15.32	10.8	21.26
rarres3	11.2	21.96	16.86	74.12	78.37	39.74	11.41	8.11	0.94
sell	5.45	0.09	0.26	45.72	42.15	6.75	4.56	43.35	43.52
grin1	4.21	23.42	11.99	15	12.75	14.97	3.08	5.82	3.38
acta1	1384.76	1077.68	1307.49	844.56	1410.93	2036	309.78	159.89	106.18
cdkn1a	51.09	36.35	36.25	49.71	59.88	38.64	17.38	18.93	17.43
fosl1	0.79	1.46	1.52	0.58	2.85	1.27	6.75	6.19	10.13
gjb3	9.07	4.74	12.19	7.06	9.39	6.63	17.03	17.75	21.07
stra6	17.38	16.47	20.35	44.9	24.56	22.73	11.35	5.08	3
trpm2	0.6	0.38	0.26	0.82	1.34	0.32	0.17	0.05	0.07
cp	726.76	570.4	416.66	240.26	217.74	466.66	555.05	586.16	760.75
tfr	79.09	143.9	140.3	70.77	135.45	156.8	238.34	351.67	699.34
chgb	1.05	4.53	4.25	3.39	6.94	3.62	1.3	0.71	1.07
nrd1	0.32	0.23	0.18	0.51	0.13	0.31	0.04	0	0.04
npy1r	7.03	5.39	9.27	12.4	13.45	9.8	5.28	2.45	3.12
lgals1	46.89	27.6	60.85	85.68	76.15	48.43	26.34	10.93	29.2
ppp3ca	2.8	9.36	5.68	8.73	6.84	6.1	1.73	2.48	3.38
mst1	100.81	98.87	52.63	52.79	65.37	105.44	165.55	195.93	166.3

Table S12. Expression pattern of differentially expressed genes (DEGs) relevant to retinoic acid synthesis and signaling during the pregnant process of *H. abdominalis*.

Gene Symbol	Gene ID	g2 (Fold Chang	P value	Comparison
aldh9a1b	ENSDARG00000037061	-2.902	0.04606	latepreg vs nonpreg
rdh10a	ENSDARG00000058730	2.158	0.0000415	latepreg vs nonpreg
cyp26a1	ENSDARG00000033999	-2.319	0.001779	midpreg vs nonpreg
rxrgb	ENSDARG00000004697	2.857	0.0000616	midpreg vs nonpreg
rxrgb	ENSDARG00000004697	1.861	0.04656	earlypreg vs nonpreg
crabp2a	ENSDARG00000040923	-1.16	0.04778	latepreg vs nonpreg
fshr	ENSDARG00000071494	-1.406	0.007188	midpreg vs nonpreg
hsd11b2	ENSDARG00000001975	-3.572	0.0003074	latepreg vs nonpreg
hsd11b2	ENSDARG00000001975	-3.152	0.0001863	midpreg vs nonpreg
hsd11b2	ENSDARG00000001975	-2.351	0.01375	earlypreg vs nonpreg
grasp	ENSDARG00000000588	-1.624	0.01904	earlypreg vs nonpreg
itgb4	ENSDARG00000028507	-1.403	0.03008	midpreg vs nonpreg
tgfa	ENSDARG00000053939	-5.856	0.03533	latepreg vs nonpreg
abcc2	ENSDARG00000014031	-1.76	0.03807	latepreg vs nonpreg
plat	ENSDARG00000062707	-2.2	0.005742	latepreg vs nonpreg
plat	ENSDARG00000062707	-1.958	0.009609	midpreg vs nonpreg
mgp	ENSDARG00000086189	-1.684	0.000028	latepreg vs nonpreg
col4a2	ENSDARG00000020781	1.217	0.02842	latepreg vs nonpreg
krt18	ENSDARG00000018404	3.176	0.007966	latepreg vs nonpreg
krt18	ENSDARG00000018404	1.224	0.02134	midpreg vs nonpreg
krt18	ENSDARG00000018404	1.379	0.01653	earlypreg vs nonpreg
col4a1	ENSDARG00000055009	1.329	0.008908	latepreg vs nonpreg
edn1	ENSDARG00000036912	3.903	0.0007164	latepreg vs nonpreg
krt4	ENSDARG00000017624	1.381	0.04903	latepreg vs nonpreg
krt4	ENSDARG00000017624	1.274	0.04448	midpreg vs nonpreg
acta2	ENSDARG00000045180	2.834	0.02364	latepreg vs nonpreg
meox1	ENSDARG00000007891	3.492	0.02379	latepreg vs nonpreg
meox1	ENSDARG00000007891	7.202	0.04448	midpreg vs nonpreg
meox1	ENSDARG00000007891	1.595	0.04963	earlypreg vs nonpreg
cetp	ENSDARG00000030872	7.602	0.0005293	midpreg vs nonpreg
apoa2	ENSDARG00000015866	6.228	0.0000012	latepreg vs nonpreg
apoa2	ENSDARG00000015866	4.774	0.0008131	midpreg vs nonpreg
nr4a3	ENSDARG00000055854	4.601	0.0008106	earlypreg vs nonpreg

Table S13. Relative quantity of annotated metabolites across different brood pouch developmental stages (Excel Table).

Table S14. Significant different metabolites (SDMs) identified for the brood pouch formation process (Excel Table).

Table S15. Significantly different metabolites (SDMs) identified for the pregnancy process (Excel Table).

Table S16. Kyoto encyclopedia of genes and genomes (KEGG) enrichment analysis of significantly different metabolites (SDMs) identified for the brood pouch formation process (Excel Table).

Table S17. Kyoto encyclopedia of genes and genomes (KEGG) enrichment analysis of significantly different metabolites (SDMs) identified for the pregnancy process (Excel Table).

Table S18. Co-enriched pathways of both transcriptomics and metabolomics for the brood pouch formation process.

PathA	PathB	PathC	Hits (Transcripts)	No. Hits (Transcripts)	Hits (Metabolites)	No. Hits (Metabolites)
Metabolism	Amino acid metabolism	Histidine metabolism	XLOC_006719	1	L-Histidine cpd:C00135; 1-Methylhistidine cpd:C01152	2
Metabolism	Amino acid metabolism	Tryptophan metabolism	H.erectus.017462	1	L-Tryptophan cpd:C00078	1
Metabolism	Carbohydrate metabolism	Amino sugar and nucleotide sugar metabolism	H.erectus.010479	1	D-Glucose cpd:C00031	1
Metabolism	Lipid metabolism	Arachidonic acid metabolism	H.erectus.008667	1	Arachidonic acid cpd:C00219; 5(S)-Hydroperoxyeicosatetraenoic acid cpd:C05356	2
Metabolism	Lipid metabolism	Primary bile acid biosynthesis	H.erectus.000227	1	Taurine cpd:C00245	1
Metabolism	Others	Purine metabolism	H.erectus.011448	1	Inosine cpd:C00294; Uric acid cpd:C00366	2
Genetic Information Processing	Others	Aminoacyl-tRNA biosynthesis	H.erectus.004816; H.erectus.009845	2	L-Phenylalanine cpd:C00079; L-Valine cpd:C00183; L-Tryptophan cpd:C00078; L-Tyrosine cpd:C00082	4

Table S19. Co-enriched pathways of both transcriptomics and metabolomics for the pregnancy process (Excel Table).

Table S20. Changes of retinoic acid, prolactin, progesterone, and testosterone across different brood pouch developmental stages.

Stage	Replicates	Muscle			
		**PRL (mIU/L)**	**PG (ng/mL)**	**T (pg/mL)**	**RA (nmol/L)**
PG	PG1	407.2874183	6.529101604	248.6522103	375.8543835
	PG4	461.3114108	7.291854716	257.3165847	381.5882977
	PG5	470.6398781	5.704263937	260.838688	594.7624861
	PG8	395.2820866	6.33989153	273.6591443	481.1035644
	PG10	357.1570467	8.020609142	243.5451605	
NF	NF1	565.3035408	12.21279485	157.8876064	549.9105349
	NF2	523.365997	12.17879616	161.1279415	585.9704843
	NF3	508.1970981	9.860972754	179.7246473	666.6275442
	NF4	518.1745022	10.08713792	162.2550146	678.3502133
	NF5	715.0457185	9.111523476	224.244034	592.3415001
	NF6	498.3008112	8.360595994	229.8793994	589.7930938
UF	UF1	641.5536204	11.98515148	162.0084673	593.2334423
	UF2	839.4793591	9.634807588	200.3641731	644.0741483
	UF3	567.9804053	13.86099198	159.3668898	731.4844849
	UF4	712.5310882	10.87058588	244.7778966	

Table S21. Correlation analysis between differentially expressed genes (DEGs) and significantly different metabolites (SDMs) identified for the brood pouch formation process (Excel Table).

Table S22. Correlation analysis between differentially expressed genes (DEGs) and significantly different metabolites (SDMs) identified for pregnancy process (Excel Table).
